# MSCA: a spectral comparison algorithm between time series to identify protein-protein interactions

**DOI:** 10.1186/s12859-015-0599-8

**Published:** 2015-05-13

**Authors:** Ailan F Arenas, Gladys E Salcedo, Andrey M Montoya, Jorge E Gomez-Marin

**Affiliations:** 1 Gepamol, Universidad del Quindío, Carrera 15 Calle 12N, Armenia, Colombia; 2 Grupo de Investigación y Asesoría en Estadística, Carrera 15 Calle 12N, 460 Armenia, Colombia

**Keywords:** Hypothesis testing, Protein-protein interactions, Time series, Toxoplasma

## Abstract

**Background:**

The interactions between pathogen proteins and their hosts allow pathogens to manipulate host cellular mechanisms to their advantage. The identification of host proteins that are targeted by virulent pathogen proteins is crucial to increase our understanding of infection mechanisms and to propose new therapeutics that target pathogens. Understanding the virulence mechanisms of pathogens requires a detailed molecular description of the proteins involved, but acquiring this knowledge is time consuming and prohibitively expensive. Therefore, we develop a statistical method based on hypothesis testing to compare the time series obtained from conversion of the physicochemical characteristics of the amino acids that form the primary structure of proteins and thus to propose potential functional relation between proteins. We called this algorithm the multiple spectral comparison algorithm (MSCA); the MSCA was inspired by the BLASTP tool and was implemented in R code. The algorithm compares and relates multiple time series according to their spectral similarities, and the biological relation between them could be interpreted as either a similar function or protein-protein interaction (PPI).

**Results:**

A simulation study showed that the MSCA works satisfactorily well when we compare unequal time series generated from ARMA processes because its power was close to 1. The MSCA presented a 70% average accuracy of detecting protein interactions using a threshold of 0.7 for our spectral measure, indicating that this algorithm could predict novel PPIs and pathogen-host interactions (PHIs) with acceptable confidence. The MSCA also was validated by its identification of well-known interactions of the human proteins MAGI1, SCRIB and JAK1, as well as interactions of the virulence proteins ROP16, ROP18, ROP17 and ROP5. We verified the spectral similarities for human intraspecific PPIs and PHIs that were previously demonstrated experimentally by other authors. We suggest that human GBP (GTPase group induced by interferon) and the CREB transcription factor family could be human substrates for the complex of ROP18, ROP17 and ROP5.

**Conclusions:**

Using multiple-hypothesis testing between the spectral densities of a set of unequal time series, we developed an algorithm that is able to identify the similarities or interactions between a set of proteins.

**Electronic supplementary material:**

The online version of this article (doi:10.1186/s12859-015-0599-8) contains supplementary material, which is available to authorized users.

## Background

The identification of protein-protein interactions (PPIs) is crucial for elucidating protein function and further understanding various biological processes in cells. Similarly, the identification of interactions between the proteins of infectious pathogens and their hosts (PHIs) may enable researchers to gain crucial insight into infection mechanisms. However, the general methodology for searching for new PPIs and PHIs, such as large-scale yeast two-hybrid approaches or coimmunoprecipitation methods, is time consuming and expensive [1]. Therefore, the design of computational tools, which can provide an efficient method of identifying potential protein interacting partners, is beneficial for minimizing the number of experiments. Current computational methods can be classified into two main approaches. The first approach is based on the genomic [2] or structural information of proteins [3,4]. However, these methods cannot be implemented when prior information about the proteins is not available. The second approach is based on protein primary sequences [5-7]. The latter type of approach is beneficial because most of the protein information in protein databases is the protein primary structure. The pattern of amino acid positions in protein primary structures give rise to an assumption that the amino acid sequence alone might be sufficient to estimate the propensity for interactions between two proteins for specific biological functions [8]. Accordingly, predicting PPIs and PHIs based only on sequence information is an ideal approach for computational techniques. Most of the methods for PPI prediction based on the primary structure information of proteins have been developed using a learning algorithm-support vector machine (SVM) combined with a kernel function to perform training and extract features from known pairs of PPIs to construct a universal model that separates positive PPIs from false PPIs [6,9-12]. Herein, we developed an algorithm to compare the spectral similarities between proteins using statistical hypothesis testing. In our case, we do not construct a general model for a protein-protein relation; instead, we compare the different spectral functions obtained using different descriptors for a query protein sequence against our own constructed database. This approach has the advantage of using only the protein primary sequence and not requiring either previous information from datasets or training.

The primary structure of proteins is a linear chain of amino acids that are each represented by one of 20 letters of the alphabet; thus, this alphabetic sequence can be translated into a numerical sequence using different physicochemical properties for each amino acid, such as the electron ion interaction potential (EIIP), hydrophobicity, polarity, polarizability, Van der Walls volume, ionization constant, and accessible solvent surface area.

However, because the distance between consecutive CA atoms in a protein sequence is 3.8*A*°, the points in some corresponding numerical sequences are considered equidistant, and the corresponding numerical sequence can be considered a time series. When two proteins are compared using some bioinformatics techniques, such as pairwise sequence alignments, the similarity between the proteins can be observed simply by looking for the amino acids that are conserved in specific positions of the two proteins. Otherwise, if we transform the same proteins into two time series, the proteins can be compared using mathematical techniques that allow us to see some hidden patterns that cannot be observed through the conventional alignment methods or motif searching patterns. However, when proteins are represented as a time series, we can use methods for extracting information from signals via spectral analysis. For instance, if there are certain periodicities or repetition patterns in two signals, prominent peaks appear in their spectra, and each one of these peaks carries relevant information that can represent either a functional or interaction relation [13-15]. This type of analysis is called an information spectrum method (ISM), and it has been successfully used to characterize protein-protein interactions between the gp120 HIV protein and its CD4, CCR5 and CXCR host cell receptors [16]. To further develop the ISM technique in this work, we propose the multiple spectral comparison algorithm (MSCA) to identify similarities between a query and our own set of proteins. The algorithm was inspired by the BLASTP tool in the sense that for each pair of proteins, we compare the spectral densities of all of their alignments rather than the amino acids themselves.

## Results and discussion

### Results

First, we determine whether MSCA can identify some interactions of the human proteins MAGI1 and SCRIB using eight different physical/chemical amino acid descriptors and four different sets of human proteins with MAGI1 and SCRIB as the query proteins. The algorithm identified the following protein relations: MAGI1-NET1 [17-19], MAGI1-FZD4 [19,20], MAGI1-ESAM [19,21], MAGI1-ABC1, MAGI1-CYSLTR2, MAGI1-ARHGAP6, MAGI1-TMEM215 and MAGI1-MARCH3 [19] with a similarity measure greater than 0.7. The MAGI1-NET1 and MAGI1-CYSLTR2 interactions were found using three different descriptors (Additional file 1: Supplementary material S1). MSCA also detected the protein-protein interactions SCRIB- *β*PIX [22], SCRIB-GLUT7, SCRIB-TANC1, SCRIB-MARCH3, SCRIB-ABC1, SCRIB-ARHGAP6, SCRIB-TAX, SCRIB-CYSLTR2 and SCRIB-TMEM215 [19]. The interactions SCRIB-MARCH3, SCRIB-TMEM215 and SCRIB- *β*PIX were also detected using three descriptors (Additional file 1: Supplementary material S2). The position and frequency of the interaction partners of MAGI1 and SCRIB did not change dramatically when we used the 4 different datasets. For this analysis, the interactions MAGI1-NET1, MAGI1-ARHGAP6, SCRIB-ARHGAP6, SCRIB-TMEM215 and SCRIB- *β*PIX appear to be the most consistent findings; these interactions exhibit the same frequency in the 4 datasets (Additional file 1: Supplementary material S1 and S2). We also evaluated JAK1 interaction partners using JAK1 as the query protein. In this case, the majority of proteins related to JAK1 were kinases, including TYK2 and SYK, which have been demonstrated to interact with JAK1 [23,24]. The most common transcription factor family was the STAT family, which are well-proven substrates for JAK1; STAT1 and STAT5b were the most frequent JAK1 partners in the 4 analyzed datasets [25] (Additional file 1: Supplementary material S3). The MSCA also found the previously known interaction JAK1-TRAF6 [26]. All of the candidates had a similarity measure greater than 0.7 for all descriptors.

Next, we assessed the pathogen-host interactions (PHIs) for some well-studied ROPKs and host proteins. The interactions between ROP16 and STATs were used to validate the MSCA, and similar to JAK1, most of the proteins related to ROP16 are kinases. ROP16 is a kinase protein that has all of the key amino acids for the phosphotransferase function [27] (Additional file 1: Supplementary material S4). Similarly, the third most frequent group that was identified to be related to ROP16 was the STAT transcription factor family; STAT5A and STAT5B were the most frequent partners, with each occurring five times (Additional file 1: Supplementary material S4). The MSCA also detected the experimentally demonstrated interactions ROP16-STAT3, ROP16-STAT6 and ROP16-STAT1 (Additional file 1: Supplementary material S4). The ROP18 have also been described that phosphorilates a member of the mouse GTPase family IRGa6 [28-30], and we also aimed to evaluate this interaction using the MSCA. The kinases were largely represented, but members of the immunity-related GTPase family (IRGs) were found 21 times (Additional file 1: Supplementary material S5). The protein ROP5 was also shown to act as a cofactor of ROP18. A recent work concluded that ROP17, ROP18 and ROP5 function as a complex and that the host substrates for ROP17 and ROP18 are members of the mouse immunity-related GTPase family [28-31]. The MSCA results for this complex showed that aside from proteins with kinase activity, the second most frequent family of proteins is the mouse IRG family, which was significantly related to ROP17 and ROP5, with 20 and 17 instances, respectively (Additional file 1: Supplementary materials S5, S6and S7).

Finally, we obtained an average accuracy of 70% for detecting protein interactions using a threshold of 0.7 for our *p*-value measure. However, the specificity of the test was improved when we increased the threshold to 0.8 (Additional file 1: Supplementary material S8). In general, we considered 70% accuracy acceptable for finding novel PPI or PHIs. Furthermore, we assumed that if the functional protein families found and described below from the sequence query with a probability higher than 0.7, the families would have some relationship with the query sequence; these proteins share spectral similarity derived from physicochemical features, and thus, this information could be interpreted as either a common functionor PPI.

### Discussion

Discovering protein interaction partners is a difficult task because it is time consuming and experimentally expensive; thus, it is necessary to generate algorithms to develop computational tools that help researchers who are deciding how to better understand the pathogen-human interaction system and decrease the number of experiments that must be performed. Previous experimental information, curated databases or 3D structural information is necessary to find potential interactions between proteins. Most of the bioinformatic programs that predict PPIs and PHIs require already characterized or experimentally proven PPIs and PHIs to transfer this information to new sequences. Therefore, our motivation was to develop a program that we called the MSCA, which will identify PPI or PHI relations between proteins from the primary sequence information itself. Each spectrum contains the information for each particular physicochemical descriptor for all of the proteins in this study. The MSCA relies on a spectral comparison of the protein sequences, but the comparison was formally designed through hypothesis testing. The MSCA confirmed all known PPIs using a similarity threshold of greater than 0.7. If a query protein has significant spectral similarities with another protein (using several descriptors) but the proteins are functionally different (in our case, we compared toxoplasma ROP kinases vs. transcription factors), this would mean that some spectral information is shared and would suggest an interaction between the proteins. When comparing series, commonality of some frequency and amplitude peaks along the spectra suggests a relation between the series. In the case of biological sequences, commonality of particular frequency peaks that arise from the periodical interaction interfaces of the proteins would suggest an interaction relationship. However, the MSCA can also identify the functional similarity (as shown in all tables). Indeed, many human kinase proteins appeared close to the ROP queries because they are also kinases. MSCA detected the human PPI between MAGI1 and SCRIB. Accordingly, in our analysis, the domains related to the G protein Rho are the third most abundant for MAGI1 and SCRIB and appeared 11 and 13 times, respectively (Additional file 1: Supplementary materials S1 and S2). The second most abundant group is the proteins related to cell-cell adhesion and integral membrane proteins. Additional experimental studies suggested that the MAGI1 and SCRIB proteins are closely associated with cell-cell adhesion and that these proteins act as scaffolds that assemble proteins close to membranes to regulate G protein Rho signaling [19,20,32,33]. Similarly, the ribosomal protein S6 kinase RPS6K and MAPK3 can interact with MAGI1 and SCRIB, respectively [34,35]. For the JAK1 validation, the transcription factor family STAT is the third most frequent family, and 10 experimental JAK-STAT interactions that had already been experimentally proven were found (Additional file 1: Supplementary material S3). Moreover, toxoplasma ROP16, ROP17 and ROP18 are grouped as active kinases, and these proteins are not highly divergent from one another [27]; however, other protein groups are also related to each ROP. Our ROP16 analysis showed that in addition to kinases, the STAT transcription factor family was represented frequently and appeared a total of 18 times (Additional file 1: Supplementary material S4). Although the experimentally proven interactions are not the most frequent, the MSCA found the ROP16-STAT3 and ROP16-STAT1 interactions one time each and found the ROP16-STAT6 twice. Following this concept, when we analyzed ROP18 and ROP17, the group with the second most frequent occurrences was the immunity-related GTPases (IRGs) (Additional file 1: Supplementary materials S5 and S6). Furthermore, the CREB human transcription factor family was identified frequently during ROP18 and ROP5 queries with 15 and 16 occurrences, respectively; CREB1 was the most frequently found member of the CREB family (Additional file 1: Supplementary materials S5 and S7). Experimental evidence also demonstrated that ROP18 interacts with the ATF6 *β* factor, which belongs to the CREB family [36]. Another human protein group related to the ROP18, ROP5, and ROP17 complex is the SMAD family, which is a group of signal transducers and transcriptional modulators that belong to the (TGF- *β*) pathway and mediate cell differentiation [37]. Finally, an interesting group that is also related to the complex ROP18/ROP17/ROP5 is the human GBP GTPase family, which consists of guanylate-binding proteins induced by interferon. These types of proteins promote inflammasome responses to pathogenic bacteria [38,39]. We consider GBPs to be possible human substrates for the ROP complex because the ROP complex is able to interact with mouse IRGs, which are also GTPases that are induced by interferon. Furthermore, human GBPs are highly induced after microbial infection and are associated with T. gondii [40]. Although the MSCA relies on spectral information methods, it compares the complete spectrum rather than only the frequency peaks that are shared among the proteins. Moreover, formal statistical testing was used. In summary, the MSCA results included highly well-known and experimentally identified PPIs as well as some new candidates that have a sound theoretical basis for an interaction. These candidates merit further experimental validation. In agreement with the accumulating evidence, the MSCA identifies some direct candidates for PPIs, PHIs and protein-function relations. At minimum, the MSCA can reduce the number of sequences in a large database that should be further studied, and the sequences that remain should be true candidates for relationships with the query protein. The MSCA provides the advantage of analyzing a large number of sequences, and the method can be generalized for any type of protein from any organism.

## Conclusions

Using multiple-hypothesis testing between the spectral densities of several time series, we developed an algorithm that can identify similarities or interactions between a set of proteins. A simulation study that compared different series generated from autoregressive moving average processes showed that the approach works satisfactorily. We also could identify some well-known interactions between proteins from a toxoplasma-host infection model. Considering the obtained accuracy, we choose a threshold of 0.70 that guarantees an interaction with the query protein.

## Methods

### Time series analysis

A time series is a set of observations {*x*_*t*_}, where each *x*_*t*_ is recorded at a different time. If the observations are recorded at discrete points, we have a discrete time series; this type of time series is used most frequently in practice. A more formal definition of a time series can be obtained using the theory of stochastic processes. In this context, the time series {*x*_*t*_, *t*=1,2,…,*T*} represents a realization of a stochastic process {*X*_*t*_, *t*∈*τ*}. Stationarity is an interesting property of stochastic processes and can be either strong or weak. Processes are strongly stationaries if their finite-dimensional distributions are time invariants, and processes are second-order stationaries when the unconditional expectations and variances are time invariants and if the correlation structures between observations *x*_*t*_ and *x*_*s*_ depend solely on the delay *k*=|*s*−*t*|.

There are two common approaches for analyzing a stationary time series depending on the domain under consideration. In the *time domain*, the analyses are largely based on the autocorrelation function (acf) given by

$$\rho_{x}(k)=\frac{\gamma_{x}(k)}{\sigma^{2}},\quad k=0,\pm 1, \pm 2,\ldots, $$

where *γ*_*x*_(*k*)=*C* *o* *v*(*x*_*t*_,*x*_*t*−*k*_)=*E*[ (*x*_*t*_−*μ*)(*x*_*t*−*k*_−*μ*)] is the autocovariance function, *μ* is the unconditional expectation and *σ* ^2^ is the unconditional variance of the process. The function *ρ*_*x*_(*k*) measures the linear dependence between pairs of observations separated by a lag *k*. If *ρ*_*x*_(*k*)=0 for all *k*≠0, the process lacks memory. In the *frequency domain*, the correlation structure is represented by the spectral density function defined as

$$f_{x}(\lambda)=\sum\limits_{k=-\infty}^{\infty} \gamma_{x}(k)\exp(-i 2\pi\lambda k),\quad \lambda\in[-1/2,1/2], $$

where *λ* is measured in cycles per unit of time. *f*_*x*_(*λ*) is the Fourier transform of *γ*_*x*_(*k*) and describes the properties of the process in terms of periodic components at different frequencies.

For a set of observations {*x*_*t*_, *t*=1,2,…,*T*}, the *discrete Fourier transform (DFT)* defined for the discrete Fourier frequencies *λ*_*j*_=*j*/*T*, *j*=0,1,2,…,[*T*/2] is given by

$$d_{x}(\lambda_{j})=\frac{1}{T}\sum\limits_{t=1}^{T} x_{t}\exp(-i 2 \pi\lambda_{j} t). $$

An estimator of the spectral density *f*_*x*_(*λ*) is the periodogram *I*_*x*_(*λ*_*j*_), which is defined as the squared modulus of the DFT,

$$I_{x}(\lambda_{j})=|d_{x}(\lambda_{j})|^{2}. $$

The value of the periodogram at each frequency represents the amount of time series variance related to this frequency or its power.

### Time series metrics

The classification and comparison of time series are problems with applications in biology, medicine, seismology, economics, and other fields, and different metrics have been proposed for classifying a set of time series. Through a simulation study, Caido et al. [41] evaluated several metrics to compare two stationary time series; most of these metrics were based on Euclidian distances. The metrics studied in the time domain were the Euclidian distance between the two time series, the two autocorrelation functions, the two partial autocorrelation functions and the Euclidian distance between their respective autoregressive parameters. In the frequency domain, the analyzed metrics were the Euclidean distance between the normalized periodograms and the Kullback-Leibler distance. A simulation study showed that distances based on the autoregressive parameters and normalized periodograms were the best metrics. Maharaj [42] proposed the *p*-value of the hypothesis testing of the equality of autoregressive parameters as a metric to compare two stationary time series. Accordingly, our algorithm uses the *p*-value of the hypothesis testing of the equality of spectral densities.

### The hypothesis testing

The issue of comparing two or more stationary time series is equivalent to evaluating whether the series were generated by the same stationary process. Stationary time series are similar if they have the same correlation structure. Coates and Diggle [43] provided some conditions for comparing two time series in the frequency domain.

Let *f*_*x*_(*λ*) and *f*_*y*_(*λ*) be the spectral density of {*x*_*t*_, *t*=1,…,*T*} and {*y*_*t*_, *t*=1,…,*T*}, respectively, and let *I*_*x*_(*λ*_*k*_) and *I*_*y*_(*λ*_*k*_) be their respective periodograms. For $k=1,\ldots,K,\ k<<T,\frac {k}{T}\approx \lambda $ and $ \frac {k}{T}\neq 0,\pm \frac {1}{2},\ldots,$ when *T*→*∞*, and when the time series are independent,

$$J(\lambda_{k})=\frac{I_{x}(\lambda_{k})}{I_{y}(\lambda_{k})}\stackrel{d}{\longrightarrow}U(\lambda)F_{2,2}, $$

where →*d* represents a distribution convergence, $\ U(\lambda)=\frac {f_{x}(\lambda)}{f_{y}(\lambda)}\ $ and *F*_2,2_ is the Fisher distribution with two degrees of freedom in both its numerator and denominator. Furthermore, $z_{k}=\ln (1+J^{-1}(\lambda _{k}))\stackrel {d}{\longrightarrow }U(\lambda _{k})\exp (1).$ Thus, when the spectral densities are equal, *U*(*λ*_*k*_)=1, and when they are asymptotically equivalent, *z*_*k*_ is exponentially distributed with a mean of 1. Consequently, the statistics $c_{j}=\sum _{k=1}^{j} z_{k}$ describe the points of a Poisson process of mean 1, and

$$\left<o_{j}=\frac{c_{j}}{c_{m}}\right>,\ \ j=1,\ldots,m=[\!T/2], $$

is a vector of the order statistics from a uniform distribution over (0,1). Then, we can test the hypothesis *H*_0_:*f*_*x*_(*λ*)=*f*_*y*_(*λ*), ∀*λ*∈(−1/2,1/2) if the statistics *o*_*j*_’s follow a uniform distribution over (0,1); for instance, we can use a Kolmogorov-Smirnov test.

### The multiple spectral comparison algorithm (MSCA)

Our approach for comparing a query and a set of proteins with unequal sizes and for sorting the proteins according to their similarities follows the steps outlined below:

Step 1: The set of proteins is transformed into a set of time series according to some amino acid properties (see properties in the Additional file 1: Supplementary material).
Step 2: Prior to each alignment between the query and a protein, hypothesis testing for equality of their spectral densities is performed and provides a *p*-value of the testing. Each alignment is understood as a match between the query and each protein using translations of order 1. The spectral similarity is represented by the mean of these *p*-values.
Step 3: Because the *p*-value from an equality testing of two time series represents a similarity measure between the two time series and satisfies the properties of a semi-metric [42], the set of proteins is sorted according to the *p*-values obtained in Step 2. The similarity between a protein and itself results in a *p*-value of 1, and this protein has the highest score. Similarities close to 1 indicate that two proteins are strongly related.

### Simulation study

Our algorithm is based on a multiple-hypothesis testing of signals of different lengths; thus, to assess its power for finite samples, we performed some simulations where we compared series generated from autoregressive moving average (ARMA) processes. {*X*_*t*_,*t*∈*τ*} is a stationary ARMA(*p*,*q*) process of zero mean if *X*_*t*_=*ϕ*_1_ *X*_*t*−1_+…+*ϕ*_*p*_ *X*_*t*−*p*_+*θ*_1_ *a*_*t*−1_+…+*θ*_*q*_ *a*_*t*−*q*_+*a*_*t*_, where the roots of the characteristic polynomials 1−*ϕ*_1_ *B*−…−*ϕ*_*p*_ *B* ^*p*^ and 1−*θ*_1_ *B*−…−*θ*_*q*_ *B* ^*q*^ are outside the unit circle and {*a*_*t*_} is a white noise process.

**Figure 1:**
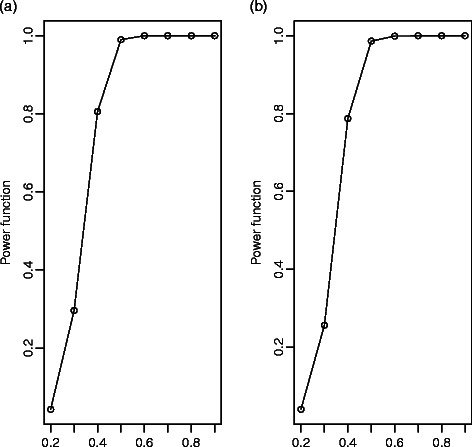
Power function for comparison of AR(1) processes **(a)** and MA(1) processes **(b)**. This figure shows the estimated power function from the hypothesis testing of Step 1.

Then, in the first case, we only compared time series AR(1) or MA(1) and generated series of length *T*=1000 from AR(1) processes with *ϕ*_1_ varying from {0.2,0.3,…,0.9}. These series were compared with series AR(1), where *ϕ*_1_=0.2 remains fixed and *T*=800. Analogously, we generated series from processes MA(1) with *θ*_1_ varying from {0.2,0.3,…,0.9} and length *T*=1000, and we compared these series with series MA(1), where *θ*_1_=0.2 remains fixed and *T*=800. In both cases, we simulated 2000 replications and considered a nominal size of 5%. We calculate this size when both signals are generated from the same process where the parameter is 0.2; in the other cases, we calculate the power. Figure 1(a) and 1(b) show the estimated power function when we compare the signals from the AR and MA processes, respectively. In general, the test performance is similar when we compare either the AR or MA time series, and the test is reasonably good because the estimated power function increases rapidly to 1 when the processes are different. In both cases, the estimated size is 0.045.

In the second case, the algorithm was used for 2000 replications of the following group of stationary time series: two time series from AR(1) process with *ϕ*_1_=0.8 and *T*=500, two time series from MA(1) process with *θ*_1_=−0.6 and *T*=400, and two time series from ARMA(1,1) process with the parameters *ϕ*_1_=0.8, *θ*_1_=−0.6 and *T*=300. In this case, we compare ARMA(1,1) with the other series. However, due to the similarity between the parameters, the series from the AR(1) or MA(1) processes could eventually be classified as similar to series from the ARMA(1,1) process, and we obtained less power due to the misclassification. The estimated size was 0.054, and the power was 0.9995 because only 1 series was misclassified.

Finally, we augmented the previous group with two time series from the AR(2) process with the parameters *ϕ*_1_=0.8, *ϕ*_2_=−0.3 and *T*=200 and with two time series from the MA(2) process with the parameters *θ*_1_=−0.6, *θ*_2_=0.3 and *T*=100. In this case, the estimated size and power were 0.048 and 0.883, respectively. The loss of power was due to 1 or 2 misclassifications with a frequency of 0.112 or 0.005, respectively, in the 2000 replications. However, for a nominal size of 10%, the estimators were 0.106 and 0.95 for size and power, respectively, with only 1 misclassified series with a frequency of 5%.

In general, the MSCA performs reasonably well because in all cases, the estimated size was close to the nominal value, and for different series, the power rapidly increased to 1, as can be expected.

### Validation study

For intra-specific (PPI) validation, we compared the different spectra for three well-studied human proteins, MAGI1, SCRIB and JAK1, against our own spectral dataset. MAGI1 (membrane-associated guanylate kinase, contains a PDZ domain), which has six PDZ domains, was found to be located to adherens and tight junctions in epithelial and endothelial cells [21,44], where MAGI1 appears to be involved in the maintenance of the junctions and in cell signal propagation. SCRIB (scribbled planar cell polarity protein), which has four PDZ domains, is known to be involved in the establishment of adherens and tight junctions as well as in the regulation of cell polarity and cell migration [45-47]. JAK1 (Janus kinase 1) is involved in the interferon *α*/ *β* and interferon *γ* signal transduction pathways. Furthermore, for (PHIs), we used the pathogen-host infection model (Toxoplasma gondii-Host). This pathogen is an obligate intracellular parasite that is able to infect any mammalian cell [48]. T. gondii is a highly successful parasite that can manipulate and control a variety of host processes due to secreted factors that interact with the host cell proteins [49-51]. Consequently, rhoptry proteins are vital for the Toxoplasma infection process and for its survival. There are a few well-documented host target proteins for toxoplasma rhoptry kinases (ROPKs) that are involved in host cell modulation. A proteomic study of rhoptry contents led to the identification of 38 rhoptry proteins [52], after a screening of a database (ToxoDB) for ROPKs revealed 44 ROPKs in the T. gondii genome using hidden Markov models (HMMs) and a phylogenomic approach [51]. ROP16 activates the STAT family transcription factors STAT1, STAT3 and STAT6 that influence the JAK/STAT pathway [53-55]. In a recent study, the authors found that ROP18 forms a complex with ROP5 and ROP17, which phosphorylate mouse immunity-related GTPase family members (IRGs) [28].

#### Datasets

The MSCA was validated by searching for protein interaction partners that had been experimentally proven for MAGI1, SCRIB and JAK1 (PPIs) and for the aforementioned interactions between ROP16, ROP18, ROP17, ROP5 and host STAT and IRG proteins (PHIs). We downloaded 930 proteins from the UniProt database (http://www.uniprot.org) and 250 kinases belonging to seven host signal transduction pathways from the KEGG database (www.genome.jp/kegg/pathway), the MAPK, JAK-STAT, NF-, TNF, HIF-1, PI3K-Akt and mTOR pathways, as well 450 transcription factors, 100 membrane proteins related to cell-cell adhesion and 130 proteins related to different activities. We mixed all of these proteins into four different sets and tested some query proteins against each group. Group 1 had 279 sequences, and the other three groups had 218 different sequences each; the queries were MAGI1 and SCRIB. For the JAK1 query, group 1 had 262 sequences, and the other three groups had 212 sequences; for ROP16, ROP18, ROP17 and ROP5, only one group of 332 proteins was considered for each validation (see Additional file 1: Supplementary material). MSCA sorted the proteins of each group according to the spectral similarity measure (the global *p*-value) of each protein with each of the following query proteins: MAGI1, SCRIB, JAK1, ROP16, ROP18, ROP17 and ROP5. (All the sequences used in the validation study were uploaded in the Additional file 2).

#### Feature conversions

PPIs can be categorized into four interaction modes: electrostatic interactions, hydrophobic interactions, steric interactions and hydrogen bonds. Here, 6 physicochemical features of amino acids were selected to transform the alphabetic sequences into a numerical series to reflect these interaction modes. These features were hydrophobicity (hydro), volume of side chains (VSC), polarity (P1), polarizability (P2), solvent accessible surface area (SASA) and the net charge index of side chains (NCISC) [56]. Furthermore, we considered 5 other physicomathematical characteristics for each amino acid, and these characteristics were successful used in the ISM technique (to look for interaction partners). These characteristics were the electron ion interaction potential (EIIP) and ionization constant (IC), which were used in [57,58] and [59], respectively. The characteristics P001, H085 and H371 were also previously proposed [60].

### Measuring the accuracy

We calculated the accuracy (ACC) and F1 scores to assess the accuracy of the MSCA. We downloaded the interaction partners for JAK1, MAGI1 and STAT3 from STRING 9.1 (string-db.org) [61]. Each protein was analyzed separately, and we designed 3 sets of negative interactions for each analysis.

## Additional files

Additional file 1 **Supplementary material.** The supplementary material contains all tables of the validation study.

Additional file 2 **Supplementary material.** The supplementary material contains all the sequences used in the validation study.
