# Supplementary material for: MSCA: a spectral comparison algorithm between time series to identify protein-protein interactions
Source: BMC Bioinformatics. 2015 May 13;16:152. doi: 10.1186/s12859-015-0599-8 (PMC4448560; doi:10.1186/s12859-015-0599-8)
Supplement: Supplementary file 1 — Supplementary material. The supplementary material contains all tables of the validation study. [file 12859_2015_599_MOESM1_ESM.pdf]

Supplementary Material S1, S2, S3, S4, S5, S6, and S7: **MSCA: A spectral comparison algorithm between time series to identify protein-protein interactions.**

Supplementary S1: Validation through MSCA for MAGI1 interactions under 8 physicochemical descriptors for 1 set of 279 sequences. Proteins are sorted according to their spectral similarity with the sequence query, similarities above 0.7 are shown. Occurrences are as a follow. Red: PPI experimentally recognized for human MAGI1 (15 times); Green: kinases; Blue: cell-cell adhesion proteins (22 times). NET1, ARHGAP6, ARHGEF16, FZD4 and CYSLTR2 are G protein related Rho signaling (11 times). NFAT: (7 times); STATs: (6 times) and CREBs: (6 times). * PPI experimentally recognized for group 1 (15 times).

The others 3 different sets had 218 sequences each one. * PPI experimentally recognized for the others groups were as a follow: group 2 (10 times); group 3 (12 times); group 4 (12 times). NET1, ARHGAP6, ARHGEF16, FZD4, CYSLTR2 and ARHGEF7 are G protein related Rho signaling. Green: kinases.

Group 1

Descriptors	HYDRO	P2	SASA	NCISC	EIIP	IC	H085	H371
Query	MAGI1	MAGI1	MAGI1	MAGI1	MAGI1	MAGI1	MAGI1	MAGI1
1	NFAT2	MAP4K4	PTK2	PI3KIBG	PI3KRB	TANC1	MAP4K4	APBA1
2	ABC1*	AFAP1	ANDR	TANC1	CD22	NFAT4	PDK11	B-raf
3	CREB1	HIF1B	MAP3K13	RAF	LRFN3	STAT2	AFAP1	PI3KIBR5
4	ATF4	NFAT2	IKKa	MAP2K2	TRAF6	HIF1B	HIF1B	Nfkappaβ
5	IKKb	MAP3K13	PI3KIBG	STAT2	SMAD2	ARHGAP6*	ACVL1	SFR
6	SP1	JAK2	HIF1B	NET1*	TRIF	PI3KIBG	STK	Ser/threN2
7	NET1*	PKNN3	SMAD4	Sgk31	PI3KB	CD166	PKAα1	Amyloid β A4
8	SFR	ATF6β	P65	PI3KRG	MADCA	TRAF5	Amyloid β A4	STK
9	CDC25B	NFAT2	ATF6β	JAK1	MAPKAPK3	B-raf	PI3KRB	SMAD3
10	MAP3K8	CADH1	ITB2	Mdm2	PI3KIBG	HIF1A	junD	TRADD
11	PAK1	ABC1*	MAP4K4	CYSLTR2*	AKT1	PITX2	STAT5B	SGK12
12	AMBP	ARHGAP6*	NFAT2	GLUT7	FZD4*	NUR77	HIF1A	PI3KIBG
13	Ser/threN2	MAP3K11	STAT2	SGK12	MAP2K4	ACTN	PKCZ2	IKKa
14	ELF4E	ADA10	PI3KD	TRAF2	ESAM*	ATF6β	CYSLTR2*	MAPKAPK3
15	FZD4*	MAP3K12	HIF1A	MAP3K3	ITB2	JunD	RPS6KA4	MAPK13
16	ICAM2	Sgk2	NET1*	MAPKAPK5	STAT5B	PI3KB	ATF6β	SMAD5
17	GLUT7	MAP3K5	Mdm2	MARCH3*	JAK3	JAM1	TRAF3	P100
18	CYSLTR2*	IKKa	PAK1	MAP3K6	E2F4	MAP3K73	MAP3K11	NFAT2
19	MADCA	ARHGEF16	PKCβ2	PAK1	NFAT2	MADCA	MAP3K1	RPS6KA4
20	CASP3	STAT5A	CADH1	AKTRACA	Amyloid β A4	PAK2	JAK1	TMEM215*

Group 2

Descriptor	HYDRO	P2	SASA	NCISC	EIIP	IC	H085	H371
Query	MAGI1	MAGI1	MAGI1	MAGI1	MAGI1	MAGI1	MAGI1	MAGI1
1	TFE2	HLTF	HLTF	TANC1	SP202	TANC1	POU2F2	SALL
2	ELF1	GTF2I	ELOA2	DMTF1	PBX1	SRBP1	AP2A	SNPC4
3	ABC1*	MITF	NFAT5	MYT1	REST	ATF7	NFYC	TRPS1
4	VEZF1	REST	ITF2	NET1*	POU6F2	ARHGAP6*	POU2F3	POU3F3
5	ATF4	SP202	MCAF1	DMRTA	OLIG2	TF3C3	TFCP2	BAZ1B
6	DMRT2	SRBP2	DMTF1	PHTF1	RUNX2	UBF1	PBX4	POU4F3
7	P5F1B	SNPC4	CARTF	CYSLTR2*	TBX4	TBX20	CYSLTR2*	PHTF1
8	TF7L1	ABC1	REST	GLUT7	TBX19	TF3C1	PHTF1	POU3F2
9	NET1*	ARHGAP6*	MITF	HSFY1	FOXE3	NFYC	TBX20	POU4F1
10	DMRTA	PHTF2	SRBP2	TFB2M	POU4F3	MTF2	ZN384	FOXC1
11	USF2	MCAF1	ELF1	BT3L2	POU5F1	MTF1	POU6F1	LMX1A
12	T2EB	T2H2L	SP202	MARCH3*	VEZF1	HSF1	AKNA	OLIG1
13	DIDO1	DMRT1	HSF1	ERCC2	DMRTA	TBX19	POU5F1	FOXS1
14	DMRT3	IRF9	BBX	TF2AA	FZD4*	ZN384	LMX1B	TFB2M
15	POU4F3	TBX2	NET1*	T2FA	DMTA2	BAZ1B	FOXF1	AP2A
16	ERCC3	ITF2	FOXN1	POU2F1	POU3F4	TBX4	TFB1M	SPDEF
17	USF1	USF2	BT3L1	GTD2A	GATA2	PHTF1	MTF2	POU5F1
18	PITX2	ARHGEF16	POU3F4	DIDO1	SRBP2	NFIB	ELF2	ELOA2
19	BAZ1B	E4F1	DMRT1	KLF1	POU5F2	POU6F1	ELF3	TF3C1
20	POU4F2	BAZ1B	USF1	TF3C4	TF3C1	NFX1	HINFP	DMTA2

Group 3

Descriptor	HYDRO	P2	SASA	NCISC	EIIP	IC	H085	H371
Query	MAGI1	MAGI1	MAGI1	MAGI1	MAGI1	MAGI1	MAGI1	MAGI1
1	RHG35	KDM4A	KDM4A	MED13	RON	TANC1	ZN521	CTND1
2	GATA6	HDAC4	HDAC4	GLI2	ZN521	EPAS1	PUF60	SP8
3	NFAC1	HDAC5	SMCA5	TANC1	GATA6	ZBED6	GATA6	NR4A2
4	E2F3	ZBT17	ZN155	TF2L1	HXB2	NR4A2	NCUG1	SP20H
5	MLXPL	RON	HDAC5	CSKP	EF1A1	ARHGAP6*	RHG35	FOXN4
6	HXA10	MGAP	MGAP	E2F6	MET	SOX7	NR2F6	ZN521
7	CTCF	NFAC1	Z280D	NET1*	ZSC31	CTND1	FOXP2	TCEA3
8	ABC1*	ABC1*	BCL6	KLF10	XRCC6	LZTL1	ATOH8	HXD13
9	PHC2	ARHGAP6*	NFAC1	CYSLTR2*	FZD4*	MGAP	CYSLTR2*	LZTL1
10	TCEA3	MED13	BARX2	GLUT7	Z280A	RBPJL	MET	EPAS1
11	FOXJ2	MYBB	NET1*	NACAD	CSKP	CSKP	LZTL1	HXA1
12	KLF5	TAF6	NOBOX	RHG35	PUF60	Z280A	NOBOX	MESP2
13	NET1*	ARHGEF16	TEAD	Z280D	ZN268	NDF1	TAB3	SP110
14	BTK	SOLH1	CTCF	ZN283	LHX8	AKND1	SALL4	SEBOX
15	SP20H	GATA4	PARP1	CHCH3	CTND1	Z280B	NRF1	RAB23
16	FX4L1	FOXO6	MED17	ZKSC3	SP110	MYB	NOC2L	NDF1
17	HXD11	SP110	TAF6	MARCH3*	NACAD	RON	ZN253	LHX8
18	HLX	SOX6	SATB1	ZN229	NFAC1	TCEA3	NECD	NFAC1
19	KLF10	BARX2	ZBT17	GABPA	RFX1	ZN229	ARHGEF7	SOX13
20	HXB2	ZBED6	HXD13	MGAP	SRCAP	FOXN3	ZSC31	TMEM215*

Group 4

Descriptors	HYDRO	P2	SASA	NCISC	EIIP	IC	H085	H371
Query	MAGI1	MAGI1	MAGI1	MAGI1	MAGI1	MAGI1	MAGI1	MAGI1
1	IRX3	WDR60	ZHX1	TANC1	STON1	TANC1	RPS6KA4	MED24
2	ESR1	SOX5	ZMYM2	RAF1	SALL3	ENC1	DEDD2	ZBTB4
3	ABC1*	MED24	BRD8	WDR11	ZN148	MED24	WDR11	NAB2
4	WDR60	ABC1*	PLK1	S6OS1	ZN226	SOX5	RD23B	KLF8
5	TEAD1	ARHGAP6*	BACH1	MED24	MED23	ARHGAP6*	STAT5B	CREM
6	NKX23	UBR5	ITCH	PFD4	IRF5	HDAC7	ZBED1	HXC10
7	SOX17	PIAS1	MDM2	NET1*	SMAD2	EZH2	MTG8	DLX3
8	HXA3	SPOP	SOX5	MDM2	ETS2	UBR5	GATA5	ETS2
9	MNDA	MTPN	TCF25	NCOR1	PLK1	HXA2	CLTR2	SOX21
10	NET1*	MED23	NR2C2	CLTR2	PIAS1	HIF1N	DLX5	ZN444
11	SOX11	BACH1	MED24	GLUT7	GATA5	NLTP	CREM	ZN226
12	ZN444	BRD8	WWP2	SALL3	DACH2	TAF5L	HXA3	ZBED1
13	COE3	ARHGEF16	EYA2	ZHX1	ZBTB4	HXB9	ARHGEF7	ZN398
14	DLX5	T22D1	SPOP	MNDA	SOX17	NR1I2	CLD18	HES1
15	HXC10	PRDM4	MCM8	MARCH3*	SOX4	RPB11	BCL10	IRX3
16	ZHX1	STAT5A	PIAS1	SDCB1	FZD4*	NR1H4	HES2	SIR1
17	TAF10	RUVB2	RPAB2	RPS6KA5	HXA3	HXD8	SOX21	CCND1
18	HXA11	REQU	NET1*	SOX30	HXD1	MNDA	HXC9	IRF5
19	ZN148	RPAB2	KPCE	ZN410	VSX1	CCND1	CDCA4	ZN148
20	FZD4*	SOX8	HDAC7	REQU	STAT5B	HXB5	PIAS1	TMEM215*

Supplementary S2: Validation through MSCA for SCRIB interactions under 8 physicochemical descriptors for 1 set of 279 sequences. Proteins are sorted according to their spectral similarity with the sequence query, similarities above 0.7 are shown. Occurrences are as a follow. Red: PPI experimentally recognized for human SCRIB (19 times); Green: kinases; Blue: cell-cell adhesion proteins (17 times). βPIX, ARHGAP6, FZD4, CYSLTR2, RAS are G protein related Rho signaling (13 times). STATs: (8); CREBs: (9). * PPI experimentally recognized (19 times).

The others 3 different sets had 218 sequences each one. * PPI experimentally recognized for the others groups were as a follow: group 2 (19 times); group 3 (18 times); group 4 (16 times). NET1, ARHGAP6, ARHGEF16, FZD4, CYSLTR2 and are G protein related Rho signaling. Green: kinases.

Group 1

Descriptors	EIIP	IC	P001	H085	H371	HYDRO	VSC	P1	P2
Query	SCRIB	SCRIB	SCRIB	SCRIB	SCRIB	SCRIB	SCRIB	SCRIB	SCRIB
1	PI3KRB	RELB	ICAM1	PTK2	LRFN3	MAP3K1	MAP4K4	MAP3K1	MAP3K5
2	TRIF	CD166	STAT3	HIF1B	IKba	PKCα*	HIF1A	TANC1*	STAT4
3	FZD4	ARHGAP6*	ERK	PKNN3	TMEM215*	PI3KIA	PKNN3	ABC1*	RELA(P65)
4	S6kinaseβ2	CEBPB	CREB1	HIF1A	R-Ras	ABC1*	STAT1	CADH1	βPIX*
5	MADCA	ANGL3	PTK2	Amyloid β A4	CEBPB	MAP4K4	JAK3	TMEM215*	JAK2
6	MAPKAPK3	TMEM215*	MAPK13	PKAα	NFAT4	SP1	PI3KIBG	MAPKAPK3	PI3KIBG
7	PTK2	PKCα*	PI3KB	PKCα*	MAP3K6	AMBP	ATF6β	SGk2	AJUBA
8	STAT1	2AAA	ABC1*	MAP3K73	PI3KD	FOS	ADA10	RAC3	CK2
9	CDC42	GLUT7*	CD22	ADA10	MEF2C	MAP3K13	forkheadboxO3	M-Ras1	ATF-6β
10	ARHGAP6*	CK2	MARCH3*	βPIX*	MAP3K5	CREB1	MAP3K1	MAPK8	MAP4K1
11	AJUBA	S6kinaseβ1	RXR	PI3KRB	ELF4B	MAP3K7	AFAP1	MARCH3*	MYC
12	CREB5D	MAP3K73	SGK2	MAP3K72	PDK1	AJUBA	CREB1	SOX2	MAP4K4
13	RAC1	AJUBA	CREB2/2	CYSLTR2*	MAPK9	SMAD9	PTK2	HIF1B	ELK4
14	ESAM	caspase-9	AFAP1	AKTRACA	E2F5	IKKb	PDK1	PI3KIBR5	STK
15	TRAF6	PKCZ2	TAX*	TRAF3	forkheadboxO3	E2F4	CREB4	ICAM2	S6kinaseβ2
16	MARCH3*	SMAD5	NFAT4	ELF4E1B	MADCA	S6kinaseβ1	MAP3K6	GAD153	caspase-9
17	NFAT-2	STAT6	CREB3B	MAP3K7	RELB	STAT1	STAT5A	RAC1	M-Ras1
18	Ser/threN2	ELF4E	JAK3	AFAP1	SMAD6	RELB	ITB2	CDKI1	STAT5B
19	MAPKAPK2	ELK4	HIF1B	MAP3K11	AKT2RACβ	ICAM2	TRIF	TIRAP	CDC25B
20	TMEM215*	ACTN1	ITB2	PKCZ2	MAPK11	CEBPB	βPIX*	Mdm2	TRAF2

Group 2

Descriptors	EIIP	IC	P001	H085	H371	HYDRO	VSC	P1	P2
Query	SCRIB	SCRIB	SCRIB	SCRIB	SCRIB	SCRIB	SCRIB	SCRIB	SCRIB
1	SP202	TFE2	ELOA2	SRBP1	NFAT5	NFX1	MCAF1	SNPC4	TF2H2
2	SNPC4	ARHGAP6*	ITF2	LMX1B	POU4F1	POU6F2	MITF	TF3C1	REST
3	FZD4	TBX20	EA3L2	MTF2	MCAF1	MYT1	KLF16	TANC1*	T2H2L
4	NFX1	SRBP2	TF2H1	REST	POU3F4	TRPS1	DMRT2	ABC1*	βPIX*
5	TCF7	TMEM215*	TF3C2	DMTF1	OLIG3	MYT1L	ARHGEF16*	SNPC2	USF2
6	GATA2	TBX18	UBF1	POU2F3	TF3C6	SALL	CARTF	ITF2	EA3L1
7	PITX2	HSF1	FOXD3	BT3L3	NFYC	IRF9	POU3F4	NFIA	TBX4
8	FOXE3	SNPC3	T2FA	TFCP2	POU3F3	ABC1*	DMRT1	MTF1	ELOA3
9	KLF13	GLUT7*	BX22	βPIX*	FOXC1	MCAF1	POU2F1	POU6F2	TF3C5
10	HLTF	TF7L2	OLIG1	COT2	TMEM215*	FOXD2	RUNX3	DMRTC	FOXD4
11	BAZ1B	KLF1	ATF5	PHTF2	DMRT1	ELF1	UBF1	DIDO1	POU4F3
12	FOXO4	BX22	SCX	ZN384	TBX18	TF3C2	ELF1	RUNX2	TF2H1
13	DMTA2	TF3C5	RUNX3	AP2A	ELYS	POU2F1	ATF5	HBP1	ITF2
14	POU6F2	HINFP	COT2	CYSLTR2*	T2EB	NFIX	DMRTB	ATF3	UBF1
15	OLIG2	BT3L2	ABC1*	POU3F2	POU4F3	POU6F1	HLTF	DMRTB	BT3L1
16	FOXC1	E4F1	HBP1	ATF6B	GT2D1	TYY1	DMTA2	SNPC1	ELF2
17	ARHGAP6*	POU6F1	DMRT3	TFB1M	POU4F2	TBX20	BT3L1	FOXE1	POU2F2
18	TBX4	NFIB	OLIG2	POU5F1	POU3F1	T2EA	TBX4	POU3F2	AP2A
19	PBX1	TAX*	TFB1M	NFYC	BT3L1	DMRT3	NKX21	USF1	KLF13
20	MARCH3*	βPIX*	MARCH3*	BT3L2	HSF4	TANC1*	POU2F3	TMEM215*	ATF5

Group 3

Descriptors	EIIP	IC	P001	H085	H371	HYDRO	VSC	P1	P2
Query	SCRIB	SCRIB	SCRIB	SCRIB	SCRIB	SCRIB	SCRIB	SCRIB	SCRIB
1	SALL4	CMIP	ZFY16	RFX1	AKND1	KAT6A	HDAC5	KAT6A	PUF60
2	EPAS1	RON	SREC	PRDM2	ZN223	PKCα*	PUF60	HDAC5	TFEB
3	SP110	ARHGAP6*	MED13	PHC2	MED13	AN30B	SP110	MGAP	βPIX*
4	FZD4	BAZ2A	KAT6A	SALL4	HXA1	VAX1	ZN711	TANC1*	RMP
5	NR4A2	HLX	RMP	SMCA5	FX4L1	MED13	FOXJ2	RON	SOX18
6	ZN112	PRDM2	GATA6	GATA6	TMEM215*	ABC1*	ARHGEF16*	AHRR	FOXR2
7	GATA6	TMEM215*	AP2E	RN19A	FOXN3	Z280D	Z280D	ABC1*	WT1
8	PKHL1	KLF5	MESP2	MET	LZTL1	SOX13	RON	FX4L5	Z280D
9	AP2E	NOBOX	MSX2	LZTL1	NCUG1	GATA6	TAF11	YAF2	CSKP
10	Z280A	GLUT7*	SOX7	PKCα*	SEBOX	NOC2L	SOX13	NACAD	RON
11	MED13	PKCα*	MK01	SP20H	DACH1	NUCG	FOXP4	PRCC	PAX2
12	FOXR2	ZN253	FOXN3	MLXPL	VAX1	SP7	Z280A	MED13	TF3B
13	ZN268	TCEA3	HXD9	FOXP2	E2F3	ZN155	PAX2	FER3L	BARX2
14	ARHGAP6*	TAF7	BAZ2A	TAB3	RN19A	ACAD	FOXP2	IRF3	HDAC4
15	RN19A	TAB3	ZN711	βPIX*	TCF21	MESP2	MED17	MSX2	MYBB
16	ZBT17	KCC1D	Z280B	NOC2L	EPAS1	GABP1	HXB3	FOXH1	ATOH8
17	HXD9	SP8	ABC1*	ZEP1	HXD13	FIZ1	EPAS1	NCUG1	CCNA1
18	ZN229	TAF9B	SOX13	CYSLTR2*	PTTG	TANC1*	SP7	E2F3	RBPJL
19	PUF60	TAF11	RHG35	HXD11	GATA6	MET	FOXR2	HXB1	CCND3
20	EF1A1	ZSC31	GSX2	HXB7	LHX8	CTND1	RMP	TMEM215*	E2F6

Group 4

Descriptor	EIIP	IC	P001	H085	H371	HYDRO	VSC	P1	P2
Query	SCRIB	SCRIB	SCRIB	SCRIB	SCRIB	SCRIB	SCRIB	SCRIB	SCRIB
1	RPB2	MTG8	HXB4	PRD14	SG3A1	T22D1	WWP2	WDR60	ADNP
2	PCNA	THOC2	MED24	βPIX*	MCM8	EDEM2	ZHX1	TANC1*	STAT4
3	STON1	ARHGAP6*	HXA2	GATA5	HES2	ZMYM2	ADNP	SOX21	THOC2
4	FZD4	NLTP	NAB2	DEDD2	TMEM215*	MEIS2	STA5A	UBR5	ZMYM2
5	HXD1	T22D1	ZN398	MTG8	PAX6	ABC1*	PLK1	ABC1*	βPIX*
6	ETS2	PAX7	DEDD2	MNDA	CDCA4	HXC10	NR2C2	TAF10	ENC1
7	ZN226	RPS6KA4	MEIS2	HXD1	NCOR1	HDAC9	ARHGEF16*	HXA6	PAX6
8	COE3	ARHGEF16*	HXD1	GIT1	ARX	ADNP	MED6	SOX14	VDR
9	MTG8	WNT16	CTAG2	CYSLTR2*	HXC10	SG3A1	SOX30	ZBTB4	MED6
10	TEAD1	NR1I2	SOX17	RPS6KA4	SOX8	DLX5	KLF3	CHRC1	GIT1*
11	CCND2	GLUT7*	BCL10	ITCH	IRF2	MTG8	T22D1	REQU	NR0B2
12	HDAC9	TEAD2	WDR60	BCL10	CTAG2	WWP2	CEBPG	COE3	IRF5
13	ARHGAP6*	ZBED1	DLX5	THOC2	GPER1	HXA3	ZBTB1	TMEM215*	PRD14
14	PPARD	ETS2	CCND1	PPARD	MDM2	MNDA	DEAF1	HXC12	TRAF1
15	SG3A1	RPB11	ABC1*	RASH	VDR	HXD8	HXC5	HXB8	T22D1
16	MARCH3*	PPARD	CHRC1	HDAC7	KLF8	CTDP1	UBR5	GSX1	RHXF1
17	NCOR1	BACH1	HXA3	IRF5	PTHY	TEAD2	TCF25	HXA9	HDAC7
18	OTX2	ENC1	OTX2	PHS	HES1	SIAH1	DLX3	CCND1	MD2L2
19	SOX17	HXB9	SOX4	VDR	TAF5L	DLX3	HXA11	HXC8	TN13B
20	APEX1	STON1	HXC11	HXA9	WDR11	JAZF1	STAT4	CEBPG	ITCH

SCRIB-GIT1 interaction was recognized in reference (41)

Supplementary S3: Validation through MSCA for JAK1 interactions under 10 physicochemical descriptors for 1 set of 262 sequences. Proteins are sorted according to their spectral similarity with the sequence query, similarities above 0.7 are shown. Occurrences are as a follow. Red: STATs family (13 times); Green: kinases; Blue: cell-cell adhesion proteins (17 times). TRAF: (6); CREBs: (6). * PPI experimentally recognized (17 times).

The others 3 different sets had 212 sequences each one. * PPI experimentally recognized for the others groups were as a follow: group 2 (14 times); group 3 (14 times); group 4 (14 times).

Group 1

Descriptors	EIIP	IC	H085	H371	HYDRO	VSC	P1	P2	SASA	NCISC
Query	JAK1	JAK1	JAK1	JAK1	JAK1	JAK1	JAK1	JAK1	JAK1	JAK1
1	NFAT-2	CD166	PTK2	ESAM	RPS6KA5	PI3KIBG	MAP3K6	PI3KIBG	MAPKAPK3	MAP3K6
2	MADCA	ELF4B	APBA1	PTK2	Nfkappaβ	MAP3K72	PTK2	MAP4K1	TRAF2	Ser/threN2
3	ANGL3	S6kinaseβ1	AKTRACA	TRADD	PKNN3	Ser/threN12	IKKb	MAP3K12	MAP3K72	TYK2*
4	STAT5A*	MAP3K6	RPS6KA5	MAP4K2	PKCα	JAK3*	Nfkappaβ	MAP3K72	Sgk12	STAT1*
5	MAP2K4	PKCα	STK	ForkheadboxO3	PI3KIBR5	AFAP1	ITB2	MAP3K7	HIF1B	2AAA
6	STAT5B*	VCAM1	PI3KIBG	SMAD5	FOS	CD166	PKNN3	HIF1B	TFDP1	PI3KIBG
7	P38	STAT6*	SYK*	STAT1*	RIP1	caspase-9	IRAK1	CD22	MST2	MAPK1
8	PAK2	SP1	TRAF3	MAPK13	Ser/threN2	MAP3K8	MAP3K5	PKNN3	NOSe	Mdm2
9	ELK1	PI3KD	MAP3K14	ACTN1	CEBPB	MAPKAPK3	STAT4	STAT4	STAT5B*	MAP3K14
10	Ser/threN12	RELB	MNK2	ELK4	STAT1*	PKNN3	MAP4K1	CREB3B	S6kinaseβ1	PI3KRG
11	2AAA	NFAT4	STAT4	PI3KD	E2F4	HIF1A	RPS6KA5	STK	PAK1	STAT2*
12	JAM1	PAK2	NFAT-2	TRAF3	APBA1	NFAT-2	PI3KRB	MAP3K2	forkheadboxO3	CREB1
13	S6kinaseβ2	CEBPB	HIF1A	MAPKAPK5	MAP3K13	CREB1	PI3KIA	CREB1	MAP4K1	ELF4E1B
14	MAPKAPK3	STAT5B*	PKAα1	CEBPB	SP1	NOSe	B-raf	MAP3K7	Ser/threN2	MAP2K2
15	Rap-1A	Amyloid β A4	SMAD9	TFDP1	CREB4	forkheadboxO3	ANDR	RELA(P65)	SMAD7	ForkheadboxO3
16	Rap-1b	CADM3	ELF4E1B	PKCgama	PI3KIA	MAP3K3	SP1	RPS6KA4	CDC25B	B-raf
17	ACTN1	AKTRACA	TYK2*	MAPKAPK3	MAP3K7	FOS	LYAM2	SMAD5	B-raf	TRAF6*
18	PI3KB	STK	MAP3K1	STK	MAP4K4	2AAA	ATF6β	CDC25B	MAP3K8	CD22
19	MAP3K7	ELK4	PKNN3	NUR77	Insulin GF	Rap-1b	MAPK8	TRAF2	PTK2	MAP3K1
20	STAT1*	ALS	CASP3	SYK*	MAP3K6	TRAF6*	ELF4E2	MYC	MAP3K12	PKAα1

Group 2

Descriptors	EIIP	IC	H085	H371	HYDRO	VSC	P1	P2	SASA	NCISC
Query	JAK1	JAK1	JAK1	JAK1	JAK1	JAK1	JAK1	JAK1	JAK1	JAK1
1	ELOA2	SRBP2	TF3C2	NFX1	DMTF1	ELF1	SRBP2	MTF1	HLTF	DMTF1
2	PIT1	TFE2	ELOA2	TF7L1	NFX1	FOXL1	TBX3	UBF1	ELOA2	TYK2*
3	STAT5A*	DIDO1	BT3L3	POU3F3	ELF4	TFCP2	ERCC3	HLTF	P63	STAT1*
4	TF3C2	SNPC3	TRPS1	DMRT1	TF3C1	JAK3*	POU3F2	TF2H1	TF3C2	ELYS
5	TBX15	GTD2B	UBF1	POU3F2	TF3C5	MITF	STAT4	TF3C5	REST	TRPS1
6	MITF	STAT6*	TF2H3	STAT1*	REST	NFIB	DMTF1	REST	TFCP2	ELOA2
7	KLF13	ABCA1	TF7L2	HINFP	CARTF	MTF1	HLTF	POU3F4	ELF4	NFAT5
8	POU6F2	ELYS	STAT4	DMTA2	STAT1*	NFIX	NKX21	ELF4	TF2AA	SRBP2
9	STAT5B*	SP202	LMX1B	KLF13	NR5A2	NKX21	MCAF1	TF2AA	MTF1	MCAF1
10	PBX1	SNPC4	NR5A2	TF3C4	GTD2A	KLF16	DMTA2	STAT5A*	STAT5B*	TF2H4
11	PITX2	TF3C5	GATA2	SPDEF	ELOA2	TF3C2	ZN384	EA3L2	NFYA	AKNA
12	COT2	POU6F2	TYK2*	LMX1B	MTF1	DMRT1	GT2D1	EA3L1	DMRT1	UBF1
13	FOXO4	BX22	NFIX	FOXN1	GATA2	DMTA2	ATF5	ERCC3	BAZ1B	STAT2*
14	TBX20	POU2F2	FOXD2	POU4F1	GTD2B	BT3L1	NFIX	FOXN1	NFIB	TBX20
15	DMTA2	SALL	NFAT5	OLIG3	DMRT3	NFIA	HSF1	PIT1	FOXL1	ABCA1
16	TFE2	TBX5	NFYC	HLTF	NFIB	STAT2*	TCFL5	TFCP2	ABCA1	TF3C1
17	OLIG2	TCFL5	COT2	FOXC1	POU6F1	JAK2	POU4F1	NKX21	CARTF	TF3C6
18	ELF2	STAT5B*	OLIG3	TFE2	P5F1B	POU5F2	TF2H1	P63	USF1	DIDO1
19	HSFY1	TF7L2	PBX1	LMX1A	DMRT2	POU2F1	DMRTA	POU2F2	ERCC3	MYT1L
20	GATA1	OVOL1	NFIA	P63	T2EA	DMRTB	GATA1	KLF13	IRF9	BT3L2

Group 3

Descriptors Query	EIIP JAK1	IC JAK1	H085 JAK1	H371 JAK1	HYDRO JAK1	VSC JAK1	P1 JAK1	P2 JAK1	SASA JAK1	NCISC JAK1
1	SP110	CTND1	ZN268	MYB	SATB1	TAF11	KDM4A	TFEB	MED17	RHG35
2	STAT5A*	Z280D	PARP1	ZBED6	GABP1	JAK3*	RHG35	HDAC4	FOXO6	NACAD
3	KDM4A	SATB1	RN19A	VAX1	PKCα	PAX2	SATB1	FOXJ2	EF1A1	RON
4	MYBB	ZSC31	NR2F6	LZTL1	MET	MED17	PHC2	FOXN4	HDAC5	ZN283
5	STAT5B*	STAT6*	SOX6	HXD13	FOXP2	PAX3	GATA4	EF1A1	MYZAP	TYK2*
6	NR2F6	RN19A	Z280A	NR4A2	CTND1	ZSC31	STAT4	SOX13	MED13	GLI2
7	EPAS1	PKCα	STAT4	STAT1*	ZN229	TAF6	KLF10	WT1	SOX13	STAT1*
8	WT1	CMIP	HXB7	HXA1	STAT1*	HDAC5	ZN268	MED17	GATA4	MED17
9	ZBT17	TEAD	MED13	PAX2	VAX1	MET	CCND3	STAT5A*	STAT5B*	ZN521
10	Z280A	MET	HXD11	NCUG1	KLF10	EGR2	FOXH1	FOXO6	ZFY16	MK01
11	AP2E	TCEA3	ZN112	SEBOX	ZN223	FOXJ2	TAF9B	ATOH8	SOLH1	MYBB
12	ZN112	ZN521	TYK2*	GATA6	HLX	EF1A1	EGR2	GATA4	MLXPL	PRDM2
13	EMX1	TAF9B	MK01	TEAD	ZN233	ZN224	RMP	FOXP2	TAF7	GABP1
14	NR4A2	HLX	AP2E	TF3B	GATA6	HXB3	ZBT17	TAF11	Z280D	ZFY16
15	KLF5	XRCC5	STAT3*	FOXN4	NOC2L	PTTG	HXC4	GABP1	TAF11	MED13
16	ZSC31	ZBED6	GATA6	XRCC5	FOXJ2	GATA4	SOX18	ZN711	HXB3	FOXP2
17	EF1A1	STAT5B*	ZFY16	RAB23	FOXN4	MYBB	AN30B	MET	TEAD	NR4A2
18	FOXR2	MYB	STAT5A*	SP8	MESP2	SOX13	EPAS1	MYBB	XRCC6	CHCH3
19	FOXJ2	CCND3	SMCA5	TCF21	TAF7	DPF1	CMIP	HXB2	MYBB	STAT2*
20	PTTG	ZN155	ATOH8	SP110	HDAC4	CHCH3	ZKSC3	HXD12	TCEA3	ZN260

Group 4

Descriptors	EIIP	IC	H085	H371	HYDRO	VSC	P1	P2	SASA	NCISC
Query	JAK1	JAK1	JAK1	JAK1	JAK1	JAK1	JAK1	JAK1	JAK1	JAK1
1	STAT5A*	T22D1	ZN226	SOX5	WWP2	NR2C2	MEIS2	WDR60	NR2C2	RPB2
2	ZBTB1	STAT6*	RPS6KA5	ETS2	RPS6KA5	WWP2	COE1	ADNP	HDAC7	ZN410
3	TR19L	CTDP1	PKCT	STAT1*	ZBTB1	PIAS1	ZN148	ENC1	WWP2	TYK2*
4	STAT5B*	BACH1	HDAC1	ZN148	T22D1	ADNP	RPS6KA1	GIT1	ADNP	S6OS1
5	MCM8	PAX7	HDAC9	KLF8	EDEM2	ZHX1	BACH1	HDAC7	ZHX1	STAT1*
6	GIT1	THOC2	ITCH	SG3A1	STAT1*	JAK3*	STAT4	STON1	HXD1	RPS6KA1
7	NR0B2	NR1I2	HXC12	NLTP	SG3A1	MED6	TEAD1	MED6	STAT5B*	EF1D
8	HDAC9	NR1I2	MNDA	HES1	SOX8	HDAC1	RPS6KA5	TN13B	GIT1	ZBTB1
9	ZN398	SOX30	BACH1	NAB2	NLTP	STON1	HXA3	BCL10	HDAC1	NR1I2
10	HXD1	BRD8	STAT4	DLX3	ZN148	REQU	ZBED1	STAT5A*	CCND1	SOX4
11	HES1	MED24	TAF5L	PPARD	PPARD	KCC1A	EZH2	NR1I2	HXC10	BRD8
12	NFE2	RPS6KA1	CCND1	SOX21	IRX3	KLF3	SOX5	NR2C2	MYCN	STON1
13	HIF1N	MTG8	TYK2*	CREM	PKCE	PKCE	ITCH	MD2L2	CEBPG	STAT2*
14	SG3A1	STAT5B*	IRF5	RUVB2	EMX2	PLK1	PKCB	RAB17	ITCH	PRKDC
15	CREM	ZHX1	HXA9	WNT16	COE3	CEBPG	BRD8	CEBPG	THOC2	ZHX1
16	IRF1	PKCE	MTG8	CCND1	SIAH1	CCND2	VSX1	REQU	MDM2	ZMYM2
17	MED23	TEAD1	RUVB2	RAB17	NR2C2	GSX1	TAF10	SOX21	NAB2	SIR6
18	HXC11	SG3A2	RASH	NFE2	MTG8	NR0B2	SOX21	RHXF1	ENC1	MDM2
19	ZN226	MDM2	STAT3*	MED24	DLX5	MED24	SOX14	HXD1	RPB4	MNDA
20	SPOP	NR1H4	COE3	MTG8	HDAC7	MD2L2	DEDD	KAD6	REQU	KLF6

Supplementary S4: Validation through MSCA for Toxoplasma ROP16 interactions under 11 physicochemical descriptors with a set of 332 sequences. Proteins are sorted according to their spectral similarity with the sequence query, similarities above 0.7 are shown. Occurrences are as a follow. Red: STATs family (18 times); Green: kinases; Blue: cell-cell adhesion proteins (36 times); yellow: IRG (11 times); brown: CREBs: (9 times); Cyan: Toxoplasma ROPs (9 times). * PHIs experimentally recognized (4 times). IRGQh (human IRG); IRGbm (mouse IRG)

DESCRIPTORS	EIIP	IC	P001	H085	H371	HYDRO	VSC	P1	P2	SASA	NCISC
QUERY	ROP16	ROP16	ROP16	ROP16	ROP16	ROP16	ROP16	ROP16	ROP16	ROP16	ROP16
1	MAP3K3	IRGQh	ADA10	Amyloid β A4	IRGb1m	STAT1*	ELF4B	ROP18	CD22	STAT5B	PKCα
2	CREB5D	TAU(MAPT)	forkheadboxO3	ROP5	IKKb	STAT2	AFAP1	MAPK8	CDC25B	LRFN3	AKT2RACβ
3	CD166	ROP11	HIF1B	Amyloid β A	TRAF2	IRGQh	CD22 m	HSF1	MAP4K1	HSF1	B-raf
4	HSF1	SMAD4	AFAP1	STAT4	forkheadboxO3	GBP2	P100	TRAF5	P100	Sgk12	IGTPm
5	MAPKAPK2	IKKb	P100	RELB	MAP3K2	IL23R	MAP4K1	ATG3	HSF1	IKKg-nemo	LYAM1
6	AJUBA	S6kinaseβ2	HSF1	RIP1	MADCA	STAT5A	TAU(MAPT)	MAP3K7	PI3KIBR5	E2F4	PKCZ2
7	TRAF5	S22A4	STAT5A	VCAM1	FOXO1	MLTK	SMAD4	CREB5D	LRFN3	STK	ELF4E1B
8	CEBPB	STK	STAT5B	ANGL3	ROP20	B-raf	TM9S1	PDK1	ROP11	ELF4B	GBP6
9	E2F5	ELK4	PKNN3	ATF6β	IRAK1	STAT5B	RELB	CASP3	MAP3K72	MNK1	MAPK1
10	SGPP1	CD166	STAT4	TRIF	PKCβ2	MAP3K73	AMRA1	CREB1	STAT5B	STAT5A	CK2
11	IIGP5	CEAM1	PI3KIA	MAP3K6	ERBB2	HIF1B	E2F4	TRADD	ATF6β	CK2	caseinkinase
12	GBP1	ATG3	Amyloid β A4	B-raf	MAPKAPK3	PKNN3	ATF6β	IRGb2m	RELA(P65)	ELK4	NFAT-2
13	ELK4	MAP2K2	ICAM1	Ser/threN2	CEBPB	MAP3K12	Amyloid β A4	IRGQh	AFAP1	TFDP1	NBR1
14	ATG10	CD22	MAP3K14	SYK	MNK1	JAK3	MNK1	AJUBA	AMRA1	SMAD4	PKAɣ
15	MAP2K6	JAM1	CADM1	STAT5B	ELF4B	Amyloid β A4	glycoskinase	ROP31	NFAT4	MAP2K2	MAP3K3
16	SOX2	ATG10	NOSe	NOSe	IKba	NOD2	STAT5A	ROP17	CREB1	MAP4K1	ROP31
17	cJUN	TRAF5	LRFN3	Sgk31	AJUBA	RPS6KA4	MAP3K2	PKAɣ	STAT5A	ROP11	NFAT2
18	HSP27	IL1B	RPTOR	NFAT-2	PI3KIA	STAT6*	MAP3K12	Sgk2	ANDR	SHLB1	S6kinaseβ2
19	ELF4E	AFAP1	STAT6*	MAPK8	RPTOR	IRGC1m	ELK4	AKTRACB2	SMAD5	RELB	PI3KD
20	LTOR1	IL23R	ICAM5	MAP2K2	STAT4	NBR1	MAP3K72	PAK2	IGTPm	MAPKAPK2	Mdm2
21	MAP3K2	S22A5	ITB2	RPTOR	ANGL3	GBP1	TRAF5	PKAα1	CK2	CREB1	2AAA
22	MAP2K1	ATG12	ATG7	ACVL1	MAP3K14	MEF2C	NFAT-4	PI3KB	PK3C3	CD22	ATG13
23	CEAM1	MAPK1	SYK	MAPK1	CADM3	MAP4K3	IKKm	IRAK4	MAP4K4	ANDR	MAP3K14
24	IRGb1m	CEBPB	MTOR	GBP5	STAT3*	JAK1	IKKg-nemo	SOX2	ELK4	IRGM2m	Sgk12
25	ICAM2	RAS precur	ser/thrN12	IRGb1m	E2F5	NFkappaB	CREB1A	AMBP	MAPK12	CADM1	PI3KRG

Supplementary S5: Validation through MSCA for Toxoplasma ROP18 interactions under 11 physicochemical descriptors with a set of 332 sequences. Proteins are sorted according to their spectral similarity with the sequence query, similarities above 0.7 are shown. Occurrences are as a follow. Green: kinases; Blue: cell-cell adhesion proteins (15 times); yellow: IRG (21 times); brown: CREBs: (15 times); gray: GBPs (10 times); purple: SMAD (11); Cyan: Toxoplasma ROPs (13 times). * PHIs experimentally recognized (20 times). (* PPIs, ROP18-ROP17)

DESCRIPTORS	EIIP	IC	P001	H085	H371	HYDRO	VSC	P1	P2	SASA	NCISC
QUERY	ROP18	ROP18	ROP18	ROP18	ROP18	ROP18	ROP18	ROP18	ROP18	ROP18	ROP18
1	TRAF6	ROP31	CEAM1	STK	ELK4	IRGb2*	AKTRACA	CREB2	TFDP1	IRGb2*	ATG13
2	ROP20	ROP20	MAP3K7	MNK2	PDCD4	SMAD1	IRGb1m*	HSF1	S6kinaseβ2	TFDP1	TM9S1
3	IRG6a*	TRAF6	GBP3	TRAF3	CK2	GBP5	MAP3K2	PI3KRG	IRGb1m*	AJUBA	MAP3K2
4	STAT4	CREB3	IKKg-nemo	AKTRACA	SHLB1	IRGC1*	STK	PAK2	Sgk2	STK	IRGC1*
5	NUR77	ANGL3	CDC25B	PKAα1	IRGM1m*	SFR	AKTRACB2	Sgk2	STK	TRAF2	CREB1
6	MAP3K3	PKCZ2	P100	PKCZ2	IRGM2m*	GBP6	MAP2K4	P53	TRAF2	IRGb1m*	ATG7
7	TRAM	RELB	MAPK10	GBP5	TFDP1	MNK2	MAP3K11	ROP16	AKTRACB2	AKTRACA	PI3KRG
8	CREB1A	TRAF5	MAPK13	PDCD4	TRAF3	SMAD5	CADH1	JAK2	ROP39	IRGM2m*	IRGQh
9	AKTRACA	ROP17*	GBP4	MEF2C	ESAM	GBP4	MAPKAPK3	CREB1	MAP3K7	ROP39	ROP39
10	STAT3	PAK2	HIF1B	caseinkinase	BECN1	CREB4	ERBB2	TYK2	HSF1	SHLB1	SP1
11	S22A4	MAPK8	P53	CREB1A	ELK1	E2F4	TFDP1	MAP4K2	MAPKAPK2	S6kinaseβ2	B-raf
12	HIF1B	IRGM1m*	IRGM2m*	MAPK1	ROP31	JAK3	PKCβ2	MAPK8	MEK1	RAF	GBP3
13	ATG10	BECN1	STK	S6kinaseβ2	caseinkinaseII	Sgk12	MAPK11	ERBB2	SMAD5	Insulin GF	IRAK1
14	FOXO1	CREB3B	ATG7	GAD153	AKT2RACB2	GBP2	IKba	PDK1	MAP3K2	MAPKAPK2	ATF6β*
15	MAP3K14	MAP3K72	RELB	AKTRACB2	MAPK13	Sgk31	caspase-9	MAP3K8	MAP3K11	HSF1	MAP3K73
16	RAC1	IIGP5	TYK11	ELF4E1B	CREB3	SQSTM	ELK1	Amyloid β A4	CADH1	MAPKAPK3	S6kinaseβ2
17	ROP39	TIRAP	ROP39	PI3KB	TRADD	PI3KIBR5	IRGM2m*	ATG13	ESAM	PKCβ2	SMAD4
18	MAP3K72	MEF2C	ITB2	ESAM	ELF4E	CREB3B	MNK1	MAPKAPK3	E2F4	Sgk2	forkheadboxO3
19	ATG5	SMAD5	S22A4	MAPK8	CREB3B	STAT5A	P38	RIP1	Insulin GF	SMAD7	Mdm2
20	GBP6	AKT2RACβ	E2F4	SMAD9	MADCA	P53	PKAPRKX	NOD2	AKT2RACβ	MEK1	MAP2K2
21	MAP2K4	STK	AFAP1	Mdm2	ELF4E1B	STAT5B	IRGb2*	IRGb2*	ELF4B	MAPK3	BCL9
22	SFR	PI3KRB	RPS6KA5	CASP3	SMAD5	RIP1	SMAD3	ROP31	IRGQh	GBP3	JAK1
23	ATG13	ACVL1	S6kinaseβ2	ELF4B	ACVL1	CREB1	Sgk2	IRGC1*	STAT2	IKba	Amyloid β A4
24	TYK11	MNK1	EVA1A	ELK4	BCL2	MAP2K2	SOX2	ADA10	IRGb2*	ESAM	MAP4K3
25	MAP3K13	IRGM2m*	TRAF6	SMAD5	SMAD9	TIRAP	ROP20	PKAα1	SRF	CADM1	sere/thrN12

Supplementary S6: Validation through MSCA for Toxoplasma ROP17 interactions under 11 physicochemical descriptors with a set of 332 sequences. Proteins are sorted according to their spectral similarity with the sequence query, similarities above 0.7 are shown. Occurrences are as a follow. Green: kinases; Blue: cell-cell adhesion proteins (24 times); yellow: IRG (20 times); brown: CREBs: (11 times); gray: GBPs (17 times); purple: SMAD (9); Cyan: Toxoplasma ROPs (9 times). * PHIs experimentally recognized (12 times). (* PPI, ROP17-ROP18 and ROP17-ROP5).

DESCRIPTORS	EIIP	IC	P001	H085	H371	HYDRO	VSC	P1	P2	SASA	NCISC
QUERY	ROP17	ROP17	ROP17	ROP17	ROP17	ROP17	ROP17	ROP17	ROP17	ROP17	ROP17
1	AKT2RACβ	GBP3	MAP3K7	PKCγ	RAF	STK	GBP7	MAP3K3	GBP2	Sgk12	AKT1RACG2
2	IL23R	TRAF5	E2F4	SP1	PAK2	AJUBA	SMAD4	STAT1	CREB1	IRGQh	P53
3	TRAF6	PKCZ2	RELA(P65)	PKCα	STAT6	GBP3	IRGQh	CREB1	GBP5	MAP3K3	ROP39
4	S6kinaseβ2	SMAD4	MAPK13	ser/threN2	ROP39	ROP39	IKKg-nemo	S22A5	Sgk2	IRGM2m*	PKCZ2
5	RELA(P65)	MAP2K2	ATG13	MAP3K7	IL23R	LRFN3	ROP11	Amyloid β A4	PKCα	AKTRACA	MAP4K1
6	IKKa	HSF1	LYAM2	PKAα1	MADCA	MAP3K7	caspase-9	NBR1	IRGQh	GBP2	GBP5
7	ESAM	TIRAP	IGTPm	STK	MAP3K3	FOS	P38	PKCα	AKT2RACβ	STK	IRG6a*
8	ICAM5	ROP18*	CREB5D	HIF1A	ANGL3	E2F4	S22A4	AFAP1	SMAD4	SMAD2	LRFN3
9	STAT6	SMAD5	PKCγ	IGTPm	SQSTM	MADCA	IIGP5	Mdm2	ALS	GBP5	SMAD3
10	IRGM1m*	ELF4E	RXR	TM9S1	MAPK11	CREB2	RAF	MAP3K6	IRGb1m*	PAK1	ELF4B
11	MAPKAPK5	CREB3B	CREB2	PKCβ2	SMAD9	CEBPB	TRAF3	IRGQh	IGTPm	IKKb	ATG3
12	AKT1RACG2	CREB1	MAP3K14	SGPP1	MAPK10	TAU(MAPT)	GBP2	B-raf	AJUBA	CREB1	PI3KIBR5
13	GBP5	MAP3K2	NUR77	IKKa	ROP20	2AAA	ELF4B	PAK1	SGPP1	GBP4	PKAγ
14	MAP3K3	Jun-D	CEAM1	MAP4K3	TRAF3	ICAM2	MLTK	PKCβ2	IRGC1*	ANGL3	TFDP1
15	TAU(MAPT)	GADD153	IRGM2m*	IL23R	TRAF5	TYK11	PKCα	GBP1	MLP3B	CADM1	S6kinaseβ1
16	ELK1	AJUBA	RIP1	B-raf	IRGb1m*	IKKg-nemo	STK	MAPKAPK3	MAP3K2	SMAD4	MAP3K72
17	SMAD5	RELA(P65)	PKAγ	PITX2	AKT2RACβ	PI3KB	IRGM1m*	MAP2K1	CDC25B	TRAF2	RELA(P65)
18	ITB2	NOD2	IRGa6*	TRAF3	STAT4	RELB	PAK1	PDK1	Sgk31	AKT1RACG2	TYK11
19	PITX2	CREB3	IIGP5h	AKTRACA	CK2	CADM3	IGTPm	IL23R	KRasb	MLTK	PI3KRG
20	ROP39	TRAF2	ESAM	ICAM5	ROP11	ICAM5	GBP4	MAP2K2	MLTK	GBP7	STAT2
21	ATG3	B-raf	JAM1	PDK1	ELK1	MAP3K72	STK	CREB2	Rap-1b	RELA(P65)	STAT1
22	ROP5*	MAPK1	CREB1	ELK4	CADM3	IRGM2m*	E2F4	TYK2	PKCγ	MAP2K1	IRGb2*
23	Rap-1A	PITX2	PDK1	GAD153	IRGM1m*	PI3KRB	ALS	MAP4K4	CASP3	ROP31	PKAβ4
24	SOX2	MAP3K3	MAPK3(ERK)	MEKK1	P53	MAP2K2	ATG7	HIF1B	GBP7	PKNN3	PAK2
25	CDC42	CEAM1	GBP2	STAT6	GBP4	AMBP	glycosykinase	ADA10	ATG3	PKCβ2	PDK1

Supplementary S7: Validation through MSCA for Toxoplasma ROP5 interactions under 11 physicochemical descriptors with a set of 332 sequences. Proteins are sorted according to their spectral similarity with the sequence query, similarities above 0.7 are shown. Occurrences are as a follow. Green: kinases; Blue: cell-cell adhesion proteins (9 times); yellow: IRG (17 times); brown: CREBs: (16 times); gray: GBPs (9 times); purple: SMAD (11); Cyan: Toxoplasma ROPs (12 times). * PHIs experimentally recognized (9 times). (* PPI, ROP5-ROP17).

DESCRIPTORS	EIIP	IC	P001	H085	H371	HYDRO	VSC	P1	P2	SASA	NCISC
QUERY	ROP5	ROP5	ROP5	ROP5	ROP5	ROP5	ROP5	ROP5	ROP5	ROP5	ROP5
1	ELK1	ANGL3	RXR	ROP39	HSF1	CREB1	CREB1	TYK11	IRGb1m*	CREB1	STK
2	CREB3B	S6kinaseβ1	STK	STK	SMAD5	STK	MAP2K4	GBP5	E2F4	IRGb1m*	IRGb2*
3	TFDP1	TRAF3	E2F4	ROP16	STK	caspase-9	SHLB1	MAP3K3	ATG13	SHLB1	PI3KB
4	CREB4	CREB3B	IRG6a*	MAP3K72	GBP4	ROP11	IRGb2*	IKKb	SHLB1	MAPKAPK3	PI3KRG
5	S6kinaseβ2	CDC25B	CREB1	AFAP1	IKKb	SMAD5	IRGM2m*	PI3KB	AKTRACB2	IRGM2m*	S22A5
6	MAP2K4	CREB1	MAPK12	GBP2	TYK11	ELF4E2	PKCβ2	2AAA	ELK1	IRGb2*	MAPK1
7	IRGM1m*	RAF	IIGP5	MAPK8	MEF2C	IRGb2*	E2F4	Sgk31	CREB3B	IKba	RIP1
8	P38	CREB3	LYAM2	CDC25B	ELK1	ELF4E	BECN1	MAPKAPK5	GBP3	MLP3B	NUR77
9	RAF	ROP31	S6	ANGL3	ROP20	ICAM2	SMAD3	MAP3K8	CREB5D	Mdm2	ATG13
10	ROP31	PI3KB	PI3KB	GBP1	TYK2	CASP3	IRAK1	MAPKAPK3	RAF	MAP2K1	RPS6KA5
11	PAK2	AKTRACA	CADM1	STAT6	MAP3K3	PDCD4	MLP3B	MYC	MLP3B	SMAD2	AKTRACB2
12	MAPK10	RELA(P65)	STAT4	SYK	MAPK10	GBP5	MAPK12	MAP2K2	IRAK4	GAD153	ROP39
13	SMAD7	PTK2	ELK4	forkheadboxO3	STAT4	IRAK4	ATG3	IGTPm	MYC	MAPK3	SP1
14	MAPK8	RPS6KA4	CREB4	TRIF	AKT2RACβ	IGTPm	ATG7	GBP2	ICAM1	bcl2	AKTRACA
15	PI3KRA4	RXR	CK2	SGPP1	SFR	MAP2K2	CREB1A	MAPK10	SMAD7	MAPK12	BCL9
16	MADCA	MAP4K2	CREB5D	2AAA	RELB	SMAD1	PKCα	RXR	Sgk2	SMAD3	ADA10
17	ROP17*	IRGM2m*	Sgk2	MAPK11	CK2	MNK2	MAPKAPK3	RAF	MAPKAPK2	ATG13	CK2
18	STAT2	SMAD5	MAPK1	NFAT2	SYK	SQSTM	SYK	ATG7	PKCɣ	MAPK1	MAP3K3
19	TRIF	STK	FOS	CADH1	TRAF3	E2F4	STMN1	RELA(P65)	CADH1	ELK1	MAP3K2
20	Rap-1A	IRGQh	MAPK13	PKCβ2	MAPK13	CREB2/2	CDKI1	MAP3K5	CASP3	MAP2K4	HIF1B
21	ROP39	Amyloid β A4	LTOR2	MAP4K4	STAT3	ROP20	CK2	MAP3K11	Mdm2	IRGMh	ATG3
22	S6	ROP20	TRAF3	MAP4K1	ELF4E	HRas1	PKAPRKX	MAP3K13	PKNN3	ROP11	HSF1
23	RPS6KA5	TRAF6	MAPK11	CREB5D	RAF	ATG13	IRGMh	PKAα1	IL1B	Sgk2	IIGP5
24	BCL2	MAPK8	MAP3K7	MLTK	ELK4	MAPK13	PDK1	PI3KD	MAPK3	RAC1	TAU(MAPT)
25	HSF1	CEBPB	cJUN	ACVL1	SMAD4	MAPK10	STK	TFDP1	Rap-1b	SMAD7	IGTPm

Supplemantary S8: MSCA interaction performance tested for 3 human proteins

Query	Interactions	*p*-value cutoff	Sensitivity	Specificity	ACC measure	F1 score
**JAK1**	67	0.80	0.58	0.83	0.70	0.67
		0.70	0.65	0.62	0.64	0.64
**MAGI1**	20	0.80	0.70	0.85	0.76	0.75
		0.70	0.65	0.80	0.72	0.70
**STAT3**	101	0.80	0.63	0.89	0.76	0.72
		0.70	0.62	0.83	0.72	0.70

	EIIP	IC	P001	H371	H085	HYDRO	VSC	P1	P2	SASA	NCISC
**Ala**	**0.0373**	**2.3**	**4.35**	**0.937**	**-0.01**	**0.62**	**27.5**	**8.1**	**0.046**	**1.181**	**0.007187**
**Cys**	**0.0829**	**1.96**	**4.65**	**1.004**	**0.12**	**0.29**	**44.6**	**5.5**	**0.128**	**1.461**	**-0.03661**
**Asp**	**0.1263**	**1.88**	**4.76**	**1.640**	**0.15**	**-0.9**	**40**	**13**	**0.105**	**1.587**	**-0.02382**
**Glu**	**0.0058**	**2.3**	**4.29**	**0.679**	**0.07**	**-0.74**	**62**	**12.3**	**0.151**	**1.862**	**0.006802**
**Phe**	**0.0946**	**1.98**	**4.66**	**0.803**	**0.03**	**1.19**	**115.5**	**5.2**	**0.29**	**2.228**	**0.037552**
**Gly**	**0.005**	**2.46**	**3.97**	**0.901**	**0.00**	**0.48**	**0**	**9**	**0**	**0.881**	**0.179052**
**His**	**0.0242**	**2.3**	**4.63**	**1.085**	**0.08**	**-0.4**	**79**	**10.4**	**0.23**	**2.025**	**-0.01069**
**Ile**	**0**	**2.4**	**3.95**	**0.178**	**-0.01**	**1.38**	**93.5**	**5.2**	**0.186**	**1.81**	**0.021631**
**Lys**	**0.0371**	**2.2**	**4.36**	**1.254**	**0.00**	**-1.5**	**100**	**11.3**	**0.219**	**2.258**	**0.017708**
**leu**	**0**	**2.4**	**4.17**	**0.808**	**-0.01**	**1.06**	**93.5**	**4.9**	**0.186**	**1.931**	**0.051672**
**Met**	**0.0823**	**2.17**	**4.52**	**0.886**	**0.04**	**0.64**	**94.1**	**5.7**	**0.221**	**2.034**	**0.002683**
**Asn**	**0.0036**	**2.2**	**4.75**	**1.080**	**0.06**	**-0.78**	**58.7**	**11.6**	**0.134**	**1.655**	**0.005392**
**Pro**	**0.0198**	**2**	**4.44**	**0.748**	**0.00**	**0.12**	**41.9**	**8**	**0.131**	**1.468**	**0.239531**
**Gln**	**0.0761**	**2.06**	**4.37**	**1.078**	**0.05**	**-0.85**	**80.7**	**10.5**	**0.18**	**1.932**	**0.049211**
**Arg**	**0.0959**	**1.82**	**4.38**	**1.725**	**0.04**	**-2.53**	**105**	**10.5**	**0.291**	**2.56**	**0.043587**
**Ser**	**0.0829**	**2.1**	**4.50**	**1.145**	**0.11**	**-0.18**	**29.3**	**9.2**	**0.062**	**1.298**	**0.004627**
**Thr**	**0.0941**	**2.09**	**4.35**	**1.487**	**0.04**	**-0.05**	**51.3**	**8.6**	**0.108**	**1.525**	**0.003352**
**Val**	**0.0057**	**2.35**	**3.95**	**0.625**	**0.01**	**1.08**	**71.5**	**5.9**	**0.14**	**1.645**	**0.057004**
**Trp**	**0.0548**	**2.37**	**4.70**	**0.803**	**0.00**	**0.81**	**145.5**	**5.4**	**0.409**	**2.663**	**0.037977**
**Tyr**	**0.0516**	**2.2**	**4.60**	**1.227**	**0.03**	**0.26**	**117.3**	**6.2**	**0.298**	**2.368**	**0.023599**

Hydrophobicity (hydro); volume of side chains (VSC); polarity (P1); polarizability (P2); solvent accessible surface area (SASA); net charge index of side chains (NCISC); Electron Ion Interaction Potential (EIIP); Ionization Constant (IC); correlated with EIIP, P001, H085 and H371 proposed in (37).

# MCSA: R code

```
remove(list=ls())
proteinas<-read.table("D:\\table.txt",fill=TRUE)
matprot<-matrix(t(proteinas),nrow=size of the first line, ncol= sequences)
mat.ser<-matprot
 n<-ncol(mat.ser)
vec.prob<-array(1,n)
seriej<-mat.ser[,n]
para=0
pos=0
  while(para==0){pos=pos+1
            if(is.na(seriej[pos])==TRUE){lj=pos-1
                                para=1}
            if(pos==length(seriej)){lj=pos
                                para=1}
             }
   seriejtrun<-seriej[1:lj]
  for(i in 1:(n-1)){
  seriei<-mat.ser[,i]
  para=0
```

```
pos=0
while(para==0){pos=pos+1
                if(is.na(seriei[pos])==TRUE){li=pos-1
                                              para=1}
                if(pos==length(seriei)){li=pos
                                          para=1}
               }
serieitrun<-seriei[1:li]
if(li<=lj){menor<-serieitrun
          mayor<-seriejtrun
          lm<-li
          lM<-lj}
if(li>lj){menor<-seriejtrun
          mayor<-serieitrun
          lm<-lj
          lM<-li}
   m<-lm/2
frec<-0:m/lm
permen<-abs(fft(menor)/sqrt(lm))^2
probac<-0
```

```
  cont<-0
  for(k in 1:(lM-lm+1)){
          seriek<-mayor[k:(k+lm-1)]
          perk<-abs(fft(seriek)/sqrt(lm))^2
          Ji<-permen[1:m]/perk[1:m]
          zi<-as.vector(log(1+1/Ji))
          sumac<-array(0,m)
          for(l in 1:m){sumac[l]<-sum(zi[1:l])}
          max<-sumac[m]
          orden<-sumac/max
          pvalor<-ks.test(orden[2:m],"punif",0,1)$p.value
          probac<-probac+pvalor
          cont<-cont+1
                      }
  vec.prob[i]<-probac/cont
        }
ord<-sort(vec.prob,decreasing=TRUE,index.return=TRUE)
salida<-cbind(ord$x,ord$ix)
print(as.data.frame(salida))
```

How to use MSCA: R code?

1. Transform your set of proteins under some descriptor.
2. Save on D drive your proteins as a table named ***table.txt*** with the largest protein in the first line of the table and query protein in the last line of the table.
3. You have to keep in mind two numbers: the **size** of the largest protein and the number of ***used sequences*** to replace them by nrow= ***size*** and ncol=***sequences,*** respectively.
4. Save the code on D drive as ***msca.txt***.
5. Install the R program in your computer.
6. Open the R program and write in the prompt the instruction source(“D:\\msca.txt”).
7. The output shows the proteins sorted according their spectral similarity with the query.
